# Evolving perceptions of treatment helpfulness across mental illnesses in Singapore: 8-year comparison using nationally representative samples

**DOI:** 10.1192/bjo.2025.10891

**Published:** 2025-11-10

**Authors:** Celeste Minn Tan, Eng Hong Tay, Shazana Shahwan, Yoke Boon Tan, Savita Gunasekaran, Bernard Chin Wee Tan, Wei Jie Ong, Weng Mooi Tan, Siow Ann Chong, Mythily Subramaniam

**Affiliations:** Research Division, Institute of Mental Health, Singapore; Ministry of Health Office for Healthcare Transformation, Singapore

**Keywords:** Register-based epidemiology, mental health services, psychological treatments, mental health literacy, help-seeking perceptions

## Abstract

**Background:**

Singapore conducted its second nationwide mental health literacy survey in 2023, following the first survey in 2015.

**Aims:**

This study aimed to ascertain the population’s beliefs about the helpfulness of treatments for mental illnesses in Singapore, and assessed changes over an 8-year period.

**Method:**

A nationally representative cohort (*n* = 4195, aged 18–67 years) was interviewed between September 2022 and February 2024, which replicated the methods of the 2015 survey (*n* = 3006, aged 18–65 years). Using a vignette-based approach, 3002 respondents rated the perceived helpfulness of 28 treatment options for alcohol abuse, dementia, depression, obsessive–compulsive disorder (OCD) and schizophrenia as either ‘helpful’ or ‘harmful’. Weighted prevalence, stratified by vignettes and logistic regressions, were performed.

**Results:**

Counselling was most frequently rated as being helpful for alcohol use disorder (94.0%) and depression (95.2%), while seeing a psychiatrist was most frequently rated helpful for schizophrenia (93.0%), dementia (85.1%) and OCD (91.6%). Across all vignettes, informal help sources, including family (80.8%) and friends (74.7%), were considered less helpful than mental health professionals, except for ‘counselling over the phone’ (58.8%) and ’seeing a general practitioner’ (69.8%). Participants in 2023 were significantly more likely to endorse psychologists, counsellors and phone counselling as being helpful than in 2015. Face-to-face help was considered more helpful than over-the-phone professional help, highlighting the continued need for a personal touch in mental health services.

**Conclusions:**

Overall, there has been an improvement in the perception of the helpfulness of mental health professionals, but targeted interventions to improve the perception of mental health services by general practitioners and institutions are essential.

Beliefs about the helpfulness of sources of treatment options for mental disorders are an important factor in help-seeking, because incorrect beliefs can hinder an individual from seeking timely, appropriate help.^1^ Positive perceptions towards evidence-based interventions have been shown to encourage both help-seeking behaviour^2^ and treatment adherence,^3^ which influences recovery outcomes.^4^ Additionally, social barriers, such as an unwillingness to seek help from friends and family,^5^ and attitudinal barriers, such as wanting to deal with problems alone, can further contribute to the gap in receiving necessary treatments for mental health problems.^6^ Given the well-established relationship between the endorsement of effective sources of help and help-seeking behaviour,^7^ it is crucial to gain an understanding of the public’s confidence in various mental health treatment options and improve their perceptions accordingly.

Singapore’s first nationwide mental health literacy study, conducted in 2014, revealed that respondents have preferences for different types of help sources based on specific disorders, which can be due to underlying beliefs about their causes.^8^ Using a vignette-based approach, results from that study also demonstrated that most respondents would recommend seeking help from friends and family for depression (54.2%), alcohol use disorder (30.1%) and schizophrenia (21.5%), while seeking help from a general practitioner (GP) was recommended most often for dementia (53.8%) and obsessive–compulsive disorder (OCD) (26.8%), when given an open-ended question on help-seeking. Findings also elucidated that the general population perceived certain self-help strategies, such as reading about how people dealt with similar problems, as ‘helpful’ more frequently than professional help for mental health and neurocognitive disorders such as OCD and dementia, respectively. Furthermore, relaxation and stress management courses were more likely to be endorsed as helpful compared with professional help for depression. As such, the study shed light on the public’s perceptions of various interventions for mental health difficulties, and showed a strong endorsement of self-help strategies and reliance on friends and family.

We anticipated significant changes in perceptions of help sources over the past 8 years due to multiple national mental health campaigns focused on improving mental health literacy. For example, the ongoing ‘Beyond the Label’ movement initiated in 2018 aimed to promote help-seeking behaviour through reducing stigma,^9^ and the ‘It’s OKAY to Reach Out’ campaign in 2021 sought to normalise mental health conversations and encourage the public to seek support.^10^ Previous population studies have also successfully discovered and tracked changes in perceptions towards mental health treatments over time. One recent meta-analysis on public attitudes towards mental health treatments has shown an encouraging increase in terms of willingness to seek help from mental health professionals, and towards medication in the past 25 years globally.^11^ However, only a handful of population-based studies have adopted a case vignette methodology to track changes in beliefs about mental health treatments using the same procedure over multiple years.^12–14^ Notably, most of these studies have mainly focused on depression and schizophrenia and there are fewer data regarding beliefs about treatments for other mental and neurocognitive disorders such as OCD or dementia. Utilising a vignette-based approach, researchers in Germany found a substantial increase in public readiness over the past 30 years to see mental health professionals such as psychiatrists and psychologists for schizophrenia and depression.^12^ Reavley and Jorm^13^ tracked changes over 16 years in Australia and found an overall increase in willingness to use antidepressants and antipsychotics to treat depression and schizophrenia, respectively. A smaller study in South Australia found a significant reduction in the perceived helpfulness of pharmacological treatments and therapists between 2004 and 2008 for depression.^14^ These results suggest that there could be fluctuations in the perception of different help sources in a nation over a number of years.

There have been yearly attempts to track the proportion of Singaporeans who are willing to seek help from healthcare professionals and informal support networks, through the national population health survey.^10^ The proportion of those willing to seek help from informal support networks fell from 79.7% in 2022 to 78.4% in 2023, while those willing to seek help from healthcare professionals increased from 56.6 in 2022 to 62.8% in 2023. However, these surveys do not report preferences for other types of help sources such as lifestyle interventions and pharmacological treatments. Neither have these population surveys in Singapore reported respondents’ preferences regarding mental health interventions according to specific mental disorders, such as OCD and alcohol use disorder.

The current study, Mind Matters 2023 (MM2023), therefore aimed to ascertain the general population’s preferences for 28 different mental health interventions based on 5 mental health and neurocognitive disorders, which were also investigated in the earlier Mind Matters study conducted in 2015 (MM2015), and to compare the changes in perceptions towards these sources of help over the 8-year interval.

## Method

### Sample

**Fig. 1:**
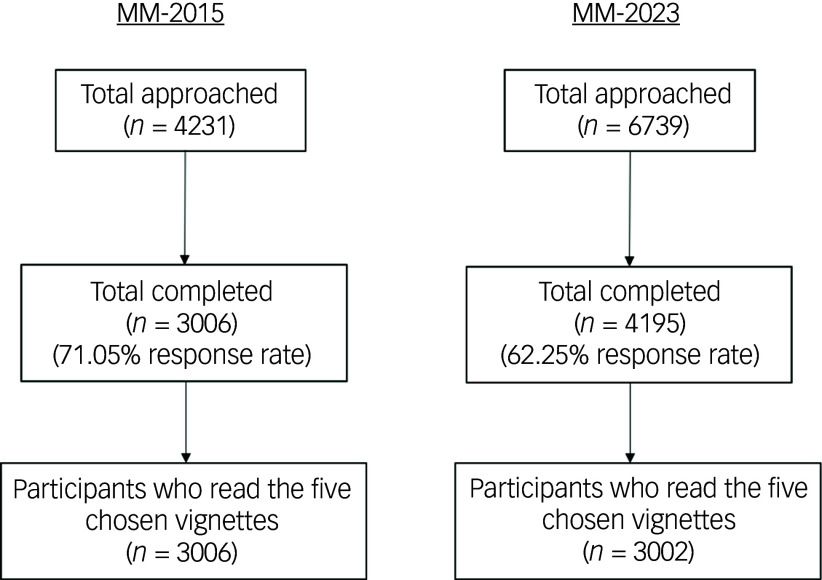
Flow diagrams of response rates for the 2015 and 2023 studies. MM2015, Mind Matters 2015; MM2023, Mind Matters 2023.

MM2023 is the second nationwide, cross-sectional survey on mental health literacy undertaken in Singapore. The present study was conducted from September 2022 to February 2024, and utilised a disproportionate stratified sampling by 12 strata (age groups: 18–34, 35–39 and 50–67 years; ethnicities: Chinese, Malay, Indian and Others) to randomly select participants from a national registry of all Singaporeans. We included Singapore citizens and permanent residents aged 18–67 years and who were able to complete the survey in one of the four official languages of Singapore (English, Chinese, Malay and Tamil). Participants with incomplete addresses, or with physical or cognitive impairments that prevented them from completing the survey, were excluded. We obtained written informed consent from legally acceptable representatives, such as parents or guardians, if the respondent was under 21 years of age. The sampling procedures and study design were similar to those of the first national mental health literacy study, MM2015, conducted from March 2014 to April 2015 among Singaporeans aged 18–65 years.^15^ However, because the sampling frames in both studies are independent and the data have been de-identified, we could not ascertain whether respondents participated in both studies. Based on the study’s aims, only participants who had read the five chosen vignettes (*n* = 3002) were included in the analysis, after excluding data from vignettes related to gambling disorder and depression with suicidal thoughts from the total 2023 sample (*n* = 4195; 62.25% response rate). All participants from the 2015 study (*n* = 3006; 71.05% response rate) read the five chosen vignettes and were included when comparing the two samples. Figure 1 illustrates how response rates and sample sizes were derived.

This study adhered to the Strengthening the Reporting of Observational Studies in Epidemiology (STROBE) guidelines.

### Measures

Face-to-face interviews were conducted either in respondents’ homes or at their preferred location by trained interviewers. This study used a vignette-based approach in which respondents were randomly assigned a vignette describing one of seven mental health/neurocognitive disorders: depression, OCD, schizophrenia, alcohol abuse, dementia, gambling disorder and depression with suicidality, with only the first five of these being the focus of this paper. We excluded gambling disorder and depression with suicidality in the current paper because these were introduced only in MM2023, and therefore we are unable to make a comparison for these disorders. The five disorders were chosen due to their prevalence^16^ and significant treatment gap in Singapore.^17^ We also ensured that the person described in the vignette was of the same ethnicity and gender as the respondent, to reduce the distance between the vignette and social reality, where respondents tend to indicate what they believe they might do rather than what they would do. All vignettes were aligned with DSM-5 criteria and developed in consultation with local clinicians to ensure that they were locally contextualised. The five vignettes are included in the supplementary materials available at https://doi.org/10.1192/bjo.2025.10891. After interviewers read the vignette aloud, respondents were asked whether they found the 28 different interventions ‘helpful’, ‘harmful’ or ‘neither’ for the person in the vignette. We categorised the interventions into four main groups: individual, medication, lifestyle and other. Sociodemographic information on age, gender and ethnicity was collected using self-reported questionnaires.

### Statistical analysis

All estimates were weighted to adjust for oversampling. Post-stratification weights were created and used in all subsequent analyses to ensure that the results were representative of the general population. Similar weighting procedures were performed for MM2015. All analyses were conducted using Stata S/E version 15.0 (StataCorp, College Station, TX, USA; https://www.stata.com/), with *P*-values <0.05 considered significant. All five disorders were combined to assess total ‘helpfulness’ ratings in the logistic regression analysis. The outcome was dichotomised into ‘helpful’ and ‘not helpful’ (i.e. ‘neither’ and ‘harmful’) for the multivariable logistic regression, for comparison of whether preferences for help sources had changed over the two studies, while controlling for age, gender, ethnicity and time points. Time point variables were used as the independent variable to determine whether there was any change in help-seeking behaviour. Weighted percentages and frequencies of ‘neither’ and ‘not helpful’ are presented in Supplementary Tables 1 and 2, respectively.

## Results

Table 1 presents the sociodemographic characteristics of the 2015 and 2023 samples;^15^ 49.1% of the 2023 sample were female and the mean age was 43.2 years (s.d. = 13.6). The weighted percentages by ethnicity were 73.8% Chinese, 13.2% Malay, 9.3% Indian and 3.7% from other ethnic groups. The 2015 sample was largely similar, with 49.1% female and a mean age of 40.9 years (s.d. = 13.4). The weighted percentages were 74.7% Chinese, 12.8% Malay, 9.1% Indian and 3.3% from other ethnic groups.

**Table 1:** Sociodemographic distribution of the samples in 2015 (*n* = 3006) and 2023 (*n* = 3002)

Sociodemographic data	MM2015 (*n* = 3006)		MM2023 (*n* = 3002)	
	Weighted, %	Unweighted, *n*	Weighted, %	Unweighted, *n*
Age groups (years)				
18–34	34.4	1152	31.5	984
35–49	35.2	896	31.6	949
50–65/67^a^	30.5	958	37.0	1069
Gender				
Female	49.1	1506	49.1	1468
Male	50.9	1500	50.9	1534
Ethnicity				
Chinese	74.7	1034	73.8	822
Malay	12.8	977	13.2	957
Indian	9.1	963	9.3	948
Other	3.3	32	3.7	275
Marital status				
Never married	31.4	927	33.4	928
Currently married	64.0	1916	59.8	1865
Separated/divorced/widowed	4.6	162	6.8	209
Highest education level				
Primary and below	13.4	431	7.5	235
Secondary/O-level/N-level	25.8	820	20.9	684
A-level/polytechnic diploma/Other diploma	31.3	999	30.3	1035
University and above	29.6	756	41.3	1048
Employment status				
Currently employed	77.6	2227	81.1	2377
Unemployed	3.9	120	3.9	130
Economically inactive	18.4	659	15.0	495
Monthly personal income (SGD)				
<2000	40.5	1346	31.6	1023
2000–5999	46.4	1162	44.9	1374
≥6000	13.1	294	23.5	567
Missing		204		60

MM2015, Mind Matters 2015; MM2023, Mind Matters 2023.

a. 50–65 years was the oldest age group for the MM2015 study, and 50–67 years for the MM2023 study.

Table 2 presents respondents’ beliefs about whether interventions were helpful for the five disorders. Across vignettes, ‘see a psychiatrist’ (90.0%) was regarded as most helpful, followed by ‘see a psychologist’ (87.6%) and then ‘see a counsellor’ (85.6%). We found that ‘see a psychiatrist’ was rated helpful most frequently for persons described with schizophrenia, OCD and dementia, while for depression and alcohol disorder, ‘see a counsellor’ was rated helpful most often. ‘Medications prescribed specifically by psychiatrists’ (84.4%) was the medication option that was most commonly rated helpful, while ‘read about people who have dealt with similar problems and how they coped’ (82.1%) was the most highly endorsed option in the ‘other’ category of interventions across vignettes.

**Table 2:** Percentage of respondents who rated help-seeking interventions as ‘helpful’ for five mental and neurocognitive disorders described in vignettes for MM2023

Intervention	Alcohol use disorder (*n* = 599)	Dementia (*n* = 599)	Depression (*n* = 602)	OCD (*n* = 600)	Schizophrenia (*n* = 602)	Total (*n* = 3002)
	%	%	%	%	%	%
Individual						
Doctor or GP	65.6	84.5	63.0	67.4	68.8	69.8
Psychiatrist	87.8	85.1	92.5	91.6	93.0	90.0
Psychologist	89.2	75.2	89.8	90.7	92.7	87.6
Social worker	68.6	64.2	73.4	56.8	82.4	69.4
Counsellor	94.0	69.9	95.2	81.1	86.9	85.6
Counselling over the phone	63.3	47.2	72.7	58.5	51.6	58.8
Close family	83.2	83.0	86.1	71.2	80.5	80.8
Close friend	72.8	72.3	84.8	67.9	75.1	74.7
Traditional Chinese medicine practitioner/Jamu/Ayurvedic-based treatment	23.6	28.8	17.6	17.2	12.6	20.1
Religious advisor	58.7	41.1	62.6	32.9	49.4	49.1
Medication						
Supplements	31.2	49.0	37.8	26.3	31.9	35.3
Tonics	25.3	35.5	28.3	17.7	22.8	26.0
Antibiotics	6.7	4.9	5.4	5.6	7.3	6.0
Sleeping pills as prescribed by a doctor	20.3	31.6	44.7	22.7	40.8	32.1
Antidepressants as prescribed by a doctor	50.9	47.0	70.9	40.0	72.2	56.5
Medicines as prescribed by a psychiatrist	85.4	82.4	83.8	79.4	90.3	84.4
Lifestyle						
Get out more, be social	39.4	68.4	69.0	59.3	69.9	61.4
Become more physically active	89.9	79.5	89.7	65.0	77.2	80.3
Yoga or meditation classes	89.3	71.9	82.7	69.4	74.7	77.8
Course on relaxation or stress management	81.3	74.9	86.7	81.9	74.9	80.1
Have an occasional drink to relax	15.5	8.1	17.3	13.8	8.6	12.7
Cut out alcohol altogether	69.4	60.5	54.1	50.1	63.1	59.5
Go on special diet	34.4	31.1	25.2	20.4	23.3	27.0
Others						
Admission to an institution	41.0	34.9	29.1	32.4	51.7	38.0
Deal with problems on his or her own	7.5	4.2	9.0	10.2	2.3	6.6
Access information from websites	58.1	51.2	51.1	60.6	44.9	53.2
Contact an expert via email or a website	63.5	62.3	59.9	70.2	54.2	62.1
Read about how people dealt with similar problems	83.5	82.1	82.0	85.8	76.2	82.1

OCD, obsessive–compulsive disorder; MM2023, Mind Matters 2023; GP, general practitioner.

Table 3 summarises respondents’ beliefs about whether interventions are harmful for the five disorders. Across vignettes, the option ‘deal with his/her problems on his/her own’ (72.5%) was viewed as the most harmful intervention, followed by ‘have an occasional alcoholic drink to relax’ (60.7%), ‘take antibiotics’ (43.3%) and ‘take sleeping pills’ (33.5%). These results were similar for all five disorders, wherein these four options were rated harmful most often. There was a low level of endorsement for some psychiatric treatments, such as taking antidepressants (15.8%) and being admitted to an institution (16.6%), with respondents finding these harmful across vignettes. Respondents also recognised the potential for information retrieved from websites as being harmful (15.3%).

**Table 3:** Percentage of respondents who rated help-seeking interventions as ‘harmful’ for five mental and neurocognitive disorders described in vignettes for MM2023

Intervention	Alcohol use disorder (*n* = 599)	Dementia (*n* = 599)	Depression (*n* = 602)	OCD (*n* = 600)	Schizophrenia (*n* = 602)	Total (*n* = 3002)
	%	%	%	%	%	%
Individual						
Doctor or GP	2.1	0.7	1.1	1.2	1.0	1.2
Psychiatrist	1.1	1.3	6.6	0.3	2.1	1.1
Psychologist	0.6	1.5	0.6	0.9	1.8	1.1
Social worker	1.4	1.9	1.2	1.3	1.2	1.4
Counsellor	0.6	1.7	0.1	1.1	1.4	1.0
Counselling over the phone	2.4	3.9	4.4	3.8	5.6	4.0
Close family	3.6	3.0	1.8	2.7	2.6	2.7
Close friend	8.4	3.7	1.5	3.3	4.2	4.2
Traditional Chinese medicine practitioner/Jamu/Ayurvedic-based treatment	11.7	13.2	14.9	16.3	20.3	15.4
Religious advisor	5.9	11.7	5.1	14.5	10.6	9.6
Medication						
Supplements	6.0	5.1	7.1	9.3	8.1	7.1
Tonics	8.0	5.9	10.6	11.9	11.2	9.5
Antibiotics	40.8	48.8	42.6	42.4	41.1	43.3
Sleeping pills as prescribed by a doctor	40.2	32.8	26.2	38.4	29.7	33.5
Antidepressants as prescribed by a doctor	16.5	19.7	10.7	21.4	10.6	15.8
Medicines as prescribed by a psychiatrist	4.3	3.9	4.3	4.6	3.5	4.1
Lifestyle						
Get out more, be social	28.0	5.0	4.2	7.0	7.7	10.4
Become more physically active	1.5	1.9	1.6	6.6	3.9	3.1
Yoga or meditation classes	1.1	2.5	1.8	2.8	4.0	2.5
Course on relaxation or stress management	1.3	0.6	2.2	0.7	1.3	1.2
Have an occasional drink to relax	60.6	62.4	56.4	58.8	65.1	60.7
Cut out alcohol altogether	12.0	4.5	11.1	6.4	7.6	8.3
Go on special diet	7.1	6.6	10.5	7.6	9.2	8.2
Others						
Admission to an institution	11.5	20.0	20.4	16.8	14.2	16.6
Deal with problems on his or her own	74.6	79.3	67.0	64.4	76.9	72.5
Access information from websites	11.0	18.3	16.7	11.6	18.7	15.3
Contact an expert via email or a website	8.7	8.4	8.2	5.5	12.6	8.7
Read about how people dealt with similar problems	4.7	0.7	2.6	2.9	2.4	2.7

OCD, obsessive–compulsive disorder; MM2023, Mind Matters 2023; GP, general practitioner.

Percentage changes in perceptions of help-seeking interventions as being helpful can be seen in Table 4. Between 2015 and 2023, there was a significant increase in the perception of the helpfulness of engaging health professionals such as psychologists (+4.6%), counsellors (+3.4%), counselling over the phone (+17.6%) and sleeping pills (+7.5%). The most striking difference was the attitude towards counselling over the phone, which was seen as helpful much more frequently in 2023 than in 2015, with a 17.6% change (*P* < 0.001). However, there was a significant decrease in the perception of the helpfulness of becoming more social (−6.2%), accessing information from websites (−6.4%) and reading about how people dealt with similar problems (−4.3%). There was no change in terms of the perception of helpfulness for certain interventions such as seeing a psychiatrist, taking medicines prescribed by a psychiatrist and becoming more physically active; endorsement of these items remained consistently high across both studies.

**Table 4:** Comparison of percentage of respondents rating interventions as ‘helpful’ between 2015 and 2023

Intervention	2015		2023			
	%	*n*	%	*n*	% change^a^	*P*-value^b^
Individual						
Doctor or GP	66.0	2120	69.8	2155	+3.8	0.060
Psychiatrist	88.5	2639	90.0	2701	+1.5	0.182
Psychologist	82.9	2476	87.6	2647	**+4.6**	<0.001
Social worker	67.6	1962	69.4	2053	+1.8	0.359
Counsellor	82.1	2491	85.6	2607	**+3.4**	0.022
Counselilng over the phone	41.2	1200	58.8	1758	**+17.6**	<0.001
Close family	83.0	2446	80.8	2441	−2.2	0.125
Close friend	78.5	2266	74.7	2257	**−3.8**	0.012
Traditional Chinese medicine practitioner/Jamu/Ayurvedic-based treatment	25.9	688	20.1	593	**−5.8**	<0.001
Religious advisor	54.0	1695	49.1	1573	−4.9	0.003
Medication						
Supplements	33.8	1183	35.3	1276	+1.5	0.639
Tonics	26.9	849	26.0	900	−0.9	0.352
Antibiotics	8.5	351	6.0	235	**−2.5**	0.002
Sleeping pills as prescribed by a doctor	24.7	775	32.1	1015	**+7.5**	<0.001
Antidepressants as prescribed by a doctor	55.1	1636	56.5	1734	+1.4	0.738
Medicines as prescribed by a psychiatrist	83.6	2484	84.4	2538	+0.8	0.598
Lifestyle						
Get out more, be social	67.6	1954	61.4	1791	**−6.2**	0.001
Become more physically active	78.6	2393	80.3	2386	+1.7	0.331
Yoga or meditation classes	72.7	2271	77.8	2388	**+5.1**	0.002
Course on relaxation or stress management	82.9	2519	80.1	2461	**−2.8**	0.024
Have an occasional drink to relax	15.4	264	12.7	268	−2.7	0.373
Cut out alcohol altogether	61.6	1311	59.5	1952	**−2.1**	0.009
Go on special diet	28.0	851	27.0	935	−1.0	0.229
Others						
Admission to an institution	40.8	1267	38.0	1221	−2.8	0.066
Deal with problems on his or her own	9.3	277	6.6	208	**−2.6**	0.006
Access information from websites	59.6	1703	53.2	1559	**−6.4**	<0.001
Contact an expert via email or a website	60.7	1713	62.1	1912	+1.3	0.328
Read about how people dealt with similar problems	86.4	2542	82.1	2468	**−4.3**	0.002

GP, general practitioner.

a. Significant percentage changes shown in bold; statistical significance was evaluated at *P* < 0.05.

b. *P*-values were derived from multivariable logistic regressions with the outcome variable (‘helpful’ versus ‘not helpful’ (reference)) while controlling for age, gender and ethnicity. The time point variable was included as a covariate to examine any significant difference in help-seeking behaviour across the two studies.

## Discussion

Drawing on Singapore’s second nationwide mental health literacy survey, this study provides population-level insights into help-seeking preferences for mental health and neurocognitive disorders and compares changes over an 8-year period. Our study found that the intervention most commonly endorsed as likely to be helpful for schizophrenia, OCD and dementia was seeing a psychiatrist, while for depression and alcohol use disorder it was seeing a counsellor. Reasons for these differences could include the frequent biological conceptualisation of mental illnesses such as schizophrenia, OCD and dementia, and the attribution of illnesses such as depression and alcohol use to weakness of character.^18,19^ Research consistently shows that more biological attributions of mental illnesses are associated with a stronger endorsement of psychiatric help.^11^ Furthermore, research supports the concept that counselling is one of the most widely recognised and essential treatment approaches in regard to managing addictions.^20^ This aligns with our findings, which show that the public perceives counsellors as the mainstay of care for alcohol addiction compared to schizophrenia and OCD.

Moreover, our comparison of attitudes towards mental health professionals in 2023 and 2015 reveals a significant increase in willingness to seek help from mental health professionals such as psychologists and counsellors. Although there was no change over the 8 years in the public’s view towards medicines prescribed by psychiatrists, the perception of their helpfulness has remained consistently high. This reflects the public’s positive perception of mental health professionals and some of their treatments. These results are also in line with the growing acceptance of consulting mental health professionals and medication, both in Singapore^10^ and globally, over the past few years.^11,12^ However, there remains a poor impression of in-patient services, because admission to institutions was perceived as being helpful by only 38.0% and harmful by 16.6% of respondents. Stigma surrounding psychiatric institutions remains high in Asian societies, and it has been reported that Singaporeans feel additional stigma for seeking mental help in such treatment settings.^21,22^ Targeted interventions to lower the level of stigma against institutionalisation are still needed, despite improvements in the perception of the helpfulness of mental health professionals. These findings, taken together, also highlight the importance of making psychiatric services more accessible in general hospitals and community settings, because many Singaporeans already have a positive perception of mental health professionals and their services.

In the area of seeking help from informal sources, our results revealed that respondents find seeking help from friends (74.7%) and family (80.8%) as being more helpful compared with some professional sources of help, such as counselling over the phone (58.5%) and contacting an expert via a website or email (62.1%), across all five disorders. Results from the study elucidated that face-to-face help was considered more helpful than remote sources of professional help. There are potential barriers to receiving professional help remotely, such as privacy, technological issues and the difficulty of maintaining a therapeutic relationship due to the less personal nature of communication.^23^ This emphasises the importance of a personal touch in mental health services today, even in the age of digital technology and advanced communication. Despite these findings, there has still been a remarkable and significant increase in the willingness to seek counselling over the phone since 2015. This could be attributed to the shift in treatments being delivered remotely during the period of lockdown due to COVID-19 in 2020, because patients were unable to physically attend psychological treatments and therapies that were labelled as non-essential services.^24^ Receiving help from mental health professionals over the phone or online remains an invaluable source of help, and it has been shown to be an effective way to alleviate symptoms.^25^ Therefore, it is essential to raise awareness about the viability of such treatment options.

Consulting a doctor or GP was seen as helpful by only a moderate proportion of respondents for all vignettes (range: 63.0–68.8%), except for dementia (84.5%). Our findings concur with previous studies showing that those living in Asia are not as likely to consult a GP for mental health difficulties compared with those in other continents, including Australia^26^ and Europe.^11^ This may be attributed to varying perspectives on the role of the GP as the initial contact in the healthcare system, and as the gatekeeper to appropriate specialists.^26^ This has important implications, because research shows that seeing a GP who can provide relevant mental health services, such as medicine, could be less stigmatising, easier to access and more cost-effective for patients with mental health difficulties than consulting a psychiatrist.^27^ Furthermore, there has been no improvement in the proportion of respondents who regard seeing a doctor or a GP as helpful in 2023 compared with 2015. The current perception towards GPs could reflect a lack of awareness and trust in the effective services provided by these professionals in Singapore. However, reliance on GPs for mental health treatment is increasingly possible following recent national healthcare reforms, such as the Healthier SG initiative that aims to ensure every individual has a dedicated family doctor.^28^ Such a model could foster a strong therapeutic alliance, built on trust and relationship, enabling more effective mental health care. Furthermore, given active efforts to involve more GPs in providing mental health services in Singapore that are in line with the national mental health and well-being strategy,^10^ these results emphasise the importance of changing the public’s perception and encouraging them to seek appropriate help from suitably equipped GPs.

Regarding interventions related to the independent acquisition of mental health information, there was a significant decrease in the perception of the helpfulness of ‘obtaining information from websites’ and ‘reading about how people dealt with similar problems’ over the study years. Only 53.2% of respondents in 2023 found accessing information from websites helpful, and 15.3% found it harmful. However, respondents still frequently identified reading about how people dealt with similar problems (82.1%) as helpful across vignettes, more so than any other lifestyle or non-medical intervention, as well as certain medical interventions. This could possibly be explained by the potential anonymity associated with gathering relevant information on personal, first-hand experiences that healthcare professionals may not always be able to provide.^29^ These results reflect the general public’s desire to access reliable mental health information and a wariness of online sources. Individuals who access mental health information online may worry about the credibility of the sources they read, and find it difficult to find relevant information.^30^ In a time where disinformation is widespread,^31^ inaccurate understanding can negatively impact the relationship between patients and health providers^32^ and affect people’s adherence to health guidelines.^33^ However, there is significant potential for internet-based sources to promote mental health positively.^29^ It is thus crucial that people can find trustworthy online sources that facilitate rather than hinder access to necessary support. Efforts can be channelled to raise awareness about credible, government-endorsed, local platforms that are already available, such as mindline.sg, a one-stop portal for mental health information.^34^ This also underscores the need for further studies to investigate how people navigate mental health information online, and to ensure that accurate and helpful information is available and accessible.

Among lifestyle interventions, becoming more physically active (80.3%) and courses on relaxation and stress management (80.1%) were most often viewed as being helpful. While perceptions of physical activity remained stable over time, this was more frequently rated helpful than many other help sources in 2023, such as antidepressants and informal help from friends and family. However, the perceived helpfulness of lifestyle interventions such as stress management and becoming more social decreased significantly over the 8 years. There is emerging evidence regarding the effectiveness of lifestyle interventions against psychopathology. Systematic reviews and meta-analyses have reported the positive impacts of lifestyle interventions, such as physical activity,^35^ stress management^36^ and social participation,^37^ on pathological symptoms including depression, anxiety and schizophrenia. Experimental evidence has also revealed that running therapy showed comparable effects on mental health, and even outperformed antidepressants in terms of the benefits to physical health in a clinical population.^38^ Given the positive perception towards these lifestyle interventions in Singapore, it is necessary to identify the key components that contribute to their effectiveness. Clear dissemination of information is then needed to enable the public to integrate these interventions into daily routines for genuine improvements in mental health.

### Limitations

Our findings must be considered in view of the study’s limitations. First, it is vital to note that the study focused on measuring perceptions towards help-seeking options rather than actual help-seeking behaviour. Nevertheless, understanding help-seeking perceptions is crucial because these significantly influence the decision to seek help. Second, because respondents were asked to indicate their perceptions on whether interventions were helpful or harmful, our results do not reflect how they might have prioritised one intervention over another. Respondents could endorse multiple options simultaneously, and these findings help to ascertain how frequently the public would find help options helpful or harmful. Lastly, this study examined only two sources of remotely administered professional help. Future studies should expand these intervention options to include services administered through video-conferencing and online chatrooms. Barriers to telemental health care services can also be examined to improve the perception and uptake of these professional services.

Strengths of this study include the use of multiple local languages to ensure inclusivity, a large sample size and a vignette-based approach that is widely accepted by researchers.^39,40^ We also ensured that the methodology remained consistent across both surveys so that we could draw a valid comparison between the two time points. This study also looked at a wide range of intervention options and mental and neurocognitive disorders that are not commonly studied in population-based vignette studies, including OCD, alcohol use disorder and dementia.

In conclusion, this nationwide study on mental health literacy helps to advance our understanding of current trends related to help-seeking perceptions in Singapore. The present study revealed a significant improvement in the perception of helpfulness of mental health professionals and the services they provide, including over-the-phone counselling services. Our findings also shed light on help sources that respondents less frequently found helpful over the study years, including accessing information from websites and being more social. This information can help us to take steps to shift perceptions of help sources in the right direction and promote help-seeking behaviour in the nation as a whole.

## Supporting information

Tan et al. supplementary material Tan et al. supplementary material

## Data Availability

The data are available on reasonable request.

## Supplementary material

The supplementary material is available online at https://doi.org/10.1192/bjo.2025.10891
